# The endometrial microbiota and early pregnancy loss

**DOI:** 10.1093/humrep/dead274

**Published:** 2024-01-09

**Authors:** Joshua Odendaal, Naomi Black, Phillip R Bennett, Jan Brosens, Siobhan Quenby, David A MacIntyre

**Affiliations:** Division of Biomedical Sciences, Clinical Sciences Research Laboratories, Warwick Medical School, Tommy's National Centre for Miscarriage Research, University of Warwick, Coventry, UK; University Hospitals Coventry & Warwickshire, Coventry, UK; Division of Biomedical Sciences, Clinical Sciences Research Laboratories, Warwick Medical School, Tommy's National Centre for Miscarriage Research, University of Warwick, Coventry, UK; University Hospitals Coventry & Warwickshire, Coventry, UK; Tommy’s National Centre for Miscarriage Research, Imperial College London, London, UK; March of Dimes Prematurity Research Centre at Imperial College London, London, UK; Imperial College Parturition Research Group, Institute of Reproductive and Developmental Biology, Department of Metabolism, Digestion and Reproduction, Imperial College London, London, UK; Division of Biomedical Sciences, Clinical Sciences Research Laboratories, Warwick Medical School, Tommy's National Centre for Miscarriage Research, University of Warwick, Coventry, UK; University Hospitals Coventry & Warwickshire, Coventry, UK; Division of Biomedical Sciences, Clinical Sciences Research Laboratories, Warwick Medical School, Tommy's National Centre for Miscarriage Research, University of Warwick, Coventry, UK; University Hospitals Coventry & Warwickshire, Coventry, UK; Tommy’s National Centre for Miscarriage Research, Imperial College London, London, UK; March of Dimes Prematurity Research Centre at Imperial College London, London, UK; Imperial College Parturition Research Group, Institute of Reproductive and Developmental Biology, Department of Metabolism, Digestion and Reproduction, Imperial College London, London, UK

**Keywords:** endometrial microbiota, immune microenvironment, miscarriage, recurrent pregnancy loss, inflammation, microbiome

## Abstract

The human endometrium is a dynamic entity that plays a pivotal role in mediating the complex interplay between the mother and developing embryo. Endometrial disruption can lead to pregnancy loss, impacting both maternal physical and psychological health. Recent research suggests that the endometrial microbiota may play a role in this, although the exact mechanisms are still being explored, aided by recent technological advancements and our growing understanding of host immune responses. Suboptimal or dysbiotic vaginal microbiota, characterized by increased microbial diversity and reduced *Lactobacillus* dominance, has been associated with various adverse reproductive events, including miscarriage. However, the mechanisms linking the lower reproductive tract microbiota with pregnancy loss remain unclear. Recent observational studies implicate a potential microbial continuum between the vaginal and endometrial niche in patients with pregnancy loss; however, transcervical sampling of the low biomass endometrium is highly prone to cross-contamination, which is often not controlled for. In this review, we explore emerging evidence supporting the theory that a dysbiotic endometrial microbiota may modulate key inflammatory pathways required for successful embryo implantation and pregnancy development. We also highlight that a greater understanding of the endometrial microbiota, its relationship with the local endometrial microenvironment, and potential interventions remain a focus for future research.

## Graphical Abstract

**Graphical Abstract:**
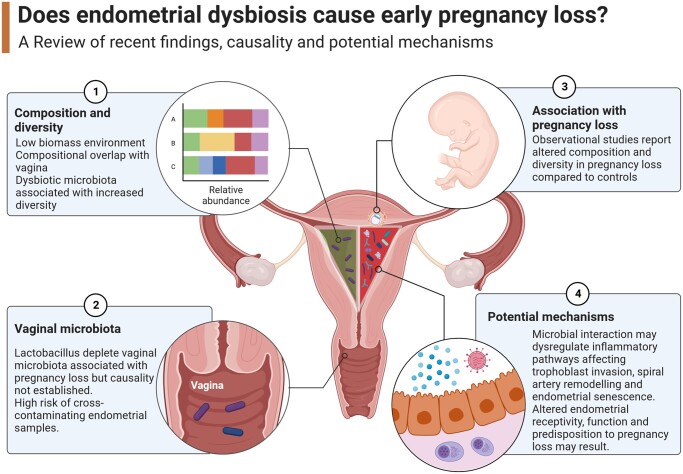
Does dysbiosis cause early pregnancy loss? A review of recent findings, causality, and potential mechanisms. Created with BioRender.com

## Introduction

The human endometrium is a dynamic tissue structure that mediates the complex interplay between the maternal host and semi-allogeneic conceptus. Disruption of this relationship can lead to miscarriage (Gellersen and Brosens, 2014) or recurrent miscarriage (RM), a condition defined by the loss of two or more pregnancies (Bender Atik *et al.*, 2018). This has devastating and long-lasting consequences for both physical and psychological health (Quenby *et al.*, 2021). The cause of miscarriage remains unclear, but key risk factors include advanced maternal age and the number of previous losses (Quenby *et al.*, 2021). While the underlying causal pathways underpinning miscarriage are complex and multifactorial, it is clear that the endometrial microenvironment plays a prominent role in early embryo–endometrial interaction (Salamonsen *et al.*, 2016). Recent efforts to characterize and quantify the constituents of the endometrial microenvironment have implicated the endometrial microbiota in RM, despite the upper reproductive tract long being considered sterile (Perez-Muñoz *et al.*, 2017). Although the human microbiome has been causally linked to a wide range of disease pathophysiology, including cardiovascular disease and diabetes mellitus (Astudillo and Mayrovitz, 2021; Lau *et al.*, 2021), the mechanism by which the microbiome shapes reproductive potential and early development is only beginning to be understood. This is partly due to improved study designs and technology platforms that facilitate more sensitive and accurate detection of the bacteria, viruses, fungi, and archaea which collectively make-up the microbiota of different niches within the reproductive tract (Marchesi and Ravel, 2015; MacIntyre *et al.*, 2017). Additionally, studies are beginning to report on the complex host immunological and inflammatory responses to specific microbiota that are consistent with emergent theories on RM.

## The lower reproductive tract microbiome

The microbiota of the lower human reproductive tract is now well described, with recent molecular characterization extending the understanding of relationships between cervicovaginal bacterial composition and states of health and disease beyond that of the long-recognized beneficial role of Lactobacilli species (France *et al.*, 2022). Dysbiosis of the vaginal microbiota, generally characterized by a loss of *Lactobacillus* species dominance and increased richness and diversity, has been associated with disease states in both non-pregnant (e.g. cervical cancer, sexually transmitted infection acquisition, and endometriosis; reviewed in Colonetti *et al.* (2023) and Ventolini *et al.* (2022)) and pregnant women (e.g. preterm premature rupture of the foetal membranes and preterm birth; reviewed in Bayar *et al.* (2020) and Bennett *et al.* (2020)). Several recent early pregnancy studies have also indicated a relationship between increased vaginal bacterial diversity and adverse early pregnancy outcomes such as miscarriage (Al-Memar *et al.*, 2020; Grewal *et al.*, 2022). For example, in comparing the vaginal microbiota of 93 women who suffered miscarriage with 74 gestational age-matched control patients who went on to deliver at term, Grewal *et al.* (2022) reported an association between *Lactobacillus* species depletion and euploid miscarriage, which was also associated with an increase in pro-inflammatory cytokines including IL-1β and IL-6. Consistent with this, Soyer Caliskan *et al.* (2022) also showed a relationship between *Lactobacillus* species-depleted vaginal microbiota communities in women with a history of RM. Despite these findings, the mechanism by which alterations in the vaginal microbiota impact events within the uterus leading to early pregnancy loss remain unclear. This has resulted in a shift in focus towards the endometrial microbiota and its potential role in shaping risk of RM.

## Endometrial microbiota composition and intra-patient variability

The uterus has long been considered a sterile environment; however, this dogma has been challenged over the last decade through the application of sensitive molecular-based approaches, including high-throughput next-generation sequencing (NGS) of bacterial 16S rRNA genes (Toson *et al.*, 2022). One of the first studies to investigate the normal microbiota using these approaches involved sampling of 58 women undergoing hysterectomy for benign conditions (Mitchell *et al.*, 2015). Using targeted quantitative PCR assays, a total of 12 common vaginal bacterial species were evaluated. At least one of these species was detected in the upper genital tract of 52/58 (90%) of the women studied with *Lactobacillus iners*, *Lactobacillus crispatus*, and *Prevotella* spp. the most commonly observed. A similar finding was demonstrated through NGS analysis of embryo transfer catheter tips collected from 70 women (some sampled more than once) undergoing IVF (Tao *et al.*, 2017). In this study, a total of 33 women had relative *Lactobacillus* species abundance >90% while 50 had relative abundance >50%. Some catheter tips were also reported to contain *Corynebacterium*, *Bifidobacterium*, *Staphylococcus*, and *Streptococcus* species. Despite a formable outer sheath being used to protect the catheter tip, such low-biomass samples are prone to contamination when sampling through the high microbial biomass cervicovaginal niche (Reschini *et al.*, 2022).

**Figure 1.**
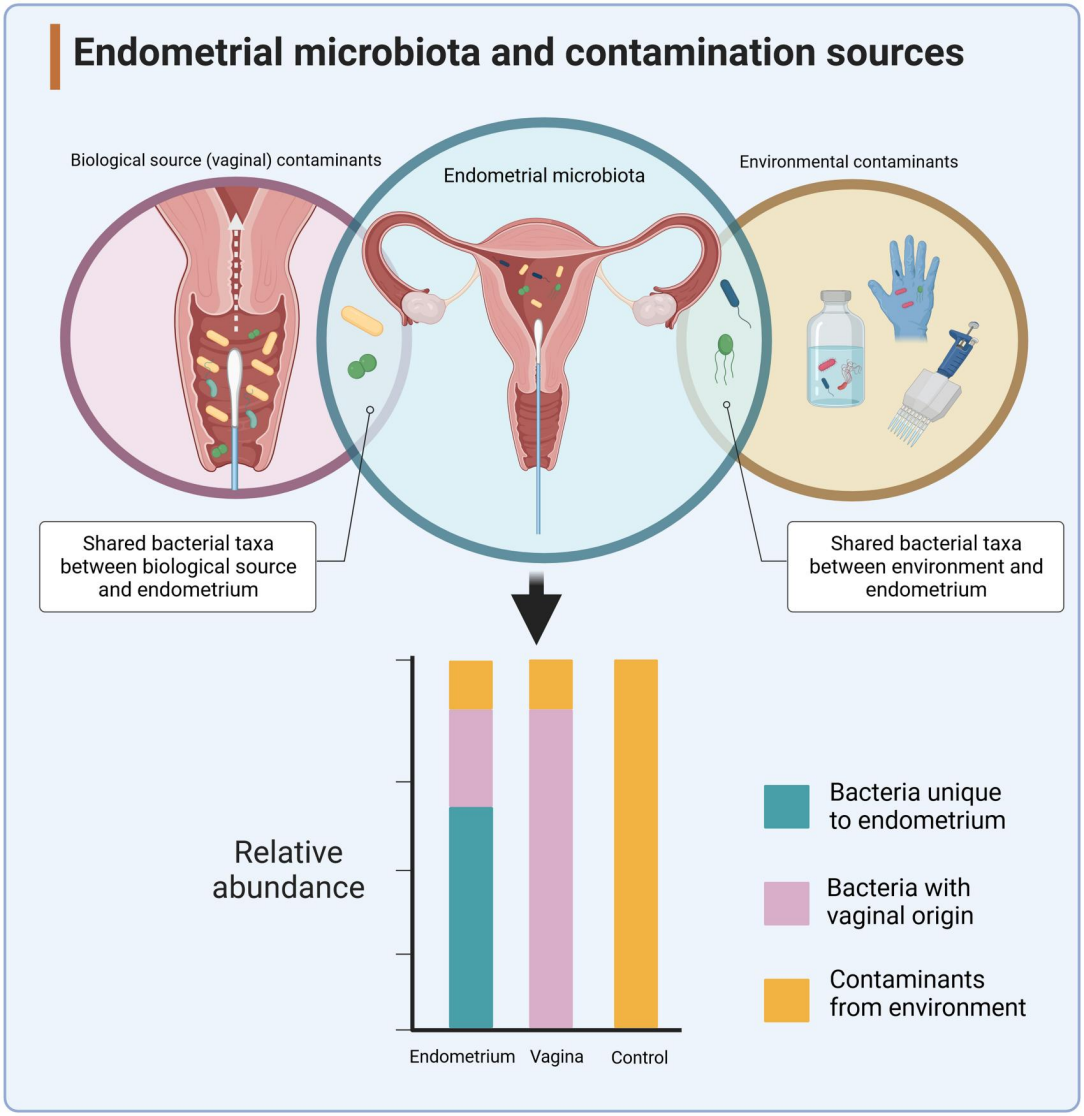
**Endometrial microbiota and contamination sources.** Accurately sampling the endometrial microbiota can be challenging owing to the high risk of cross-contamination. Sampling through the cervicovaginal route is the most practical but risks contamination from bacteria resident in the vagina, many of which are likely also present in the endometrium. Bacterial DNA may exist on protective equipment present in both the clinical and laboratory environments, as well as in laboratory reagents. Owing to the low biomass of the endometrial microbiota, the PCR amplification and sequencing strategies may lead to significantly confounded results. Created with BioRender.com.

The potential for cross-contamination during sampling procedures makes it challenging to assess to what extent a microbial continuum may exist between the cervicovaginal and endometrial mucosa. Analysis of matched endometrial and vaginal microbiota profiles collected from a small number of women undergoing hysterectomy (n = 10) demonstrated broad similarities in the composition of the microbiota across the female reproductive tract (Miles *et al.*, 2017). However, other studies have indicated that the endometrial microbiota in many women is compositionally unique and distinct from the vagina. For example, in a study of 13 fertile women who provided 26 pairs of matched vaginal and endometrial fluid aspirates, 6 paired samples were reported to have completely different bacterial profiles (Moreno *et al.*, 2016). A larger-scale study by Chen *et al.* (2017) assessed microbiota compositional profiles of 110 women across six sites along the female reproductive tract. As previously reported, *Lactobacillus* species were the dominant taxa within the vaginal microbiota; yet, this dissipated as samples progressed into the endometrium and upper reproductive tract. Differing degrees of correlation between the lower and upper reproductive tract microbiota across different patient populations may reflect health and disease phenotypes. However, the heterogeneity in findings across studies also likely highlights the challenges of obtaining contamination-free endometrial samples. As described in Fig. 1, an endometrial sample with no existing microbial biomass is highly prone to contamination during sampling or contamination from the environment (e.g. kit reagents, surgical and lab environments), which, following amplification and sequencing, could erroneously indicate the existence of a microbiota profile similar to that of vaginal taxa or environmental contaminants. Careful study design that permits assessment and correction for such potential sources of confounding is critical, yet often omitted from studies purporting to show the existence of an endometrial microbiome. Key considerations on study design have previously been reviewed (Molina *et al.*, 2021).

In addition to interpatient variability, across-cycle microbiota changes have been postulated. Sola-Leyva *et al.* (2021), sequenced 14 paired endometrial samples taken during the proliferative and mid-secretory phases from healthy controls. Significant across-cycle differences in the abundance of non-*Lactobacilli* species were demonstrated. However, in contrast to these findings, Kyono *et al.* (2018) reported a stable relative abundance of *Lactobacillus* species across the menstrual cycle.

## The association between the endometrial microbiota and miscarriage

A summary of the studies assessing the relationship between the endometrial microbiota and miscarriage is presented in Table 1. As is evident, these studies have used a variety of methodological and analytical approaches and have examined different types of miscarriages.

**Table 1.** Summary of studies assessing the human endometrial microbiota in relation to miscarriage.

Author/year	Population size/type	Sampling method	Sample timing	Method used for microbiota characterisation	Sequencing control	Reported parameters of microbiota	Findings
Liu *et al.* (2022)	25 women with RPL 25 healthy controls	Uterine lavage fluid using 2 ml of sterile saline and sheathed tube to pass through cervical canal Endometrial tissue using sheathed negative pressure catheter for suction and curettage of endometrium	5–7 days after LH surge	16S rRNA sequencing of V3–V4 region	Negative controls: normal saline, transfer medium	Chao1, Shannon index Bray–Curtis Relative abundance	*No difference in Chao1 or Shannon index.* *Beta diversity significantly higher in miscarriage uterine lavage than controls but not endometrial tissue.* *Decreases in gamma interferon, IL-6 in miscarriage group*
Masucci *et al.* (2023)	40 women with RPL (15 genetically susceptible to coeliac disease) 7 healthy controls	Tao Brush endometrial sampler	Cycle Days 19–24	16S rRNA sequencing of V3–V4 region	Not reported	Shannon index Pielou’s evenness Weighted UniFrac distances Relative abundance	*Increased microbial diversity in controls.* *Reduced Lactobacillus acidophilus in RPL group but increased Lactobacillus iners*
Moreno *et al.* (2022)	27 women with biochemical pregnancy after IVF 28 women with clinical miscarriage after IVF 141 women with live birth after IVF	Endometrial fluid aspirated with sterile embryo transfer catheter Endometrial biopsy with Cornier cannula	Cycle prior to embryo transfer, sample after 5 days progesterone	16S rRNA sequencing of V2–4–8 and V3–6, 7–9 regions	Blank samples: RNA later Positive control: *E. coli* DNA Negative control: nuclease-free water	Relative abundance	*Lactobacillus enriched in live birth* *Enterococcus, Klebsiella and Staphylococcus enriched in pregnancy loss*
Peuranpää *et al.* (2022)	47 women with RPL 39 health controls	Pipelle endometrial biopsy	6–8 days after LH surge	16S rRNA sequencing of V3–V4 region (Internal transcribed spacer 1 amplicon sequencing of fungal ITS-1 region)	Negative controls used Positive controls from earlier project re-sequenced	Relative abundance PERMANOVA analysis	*Lactobacillus crispatus more abundant in healthy controls* *Gardnerella vaginalis more abundant in RPL group*
Shi *et al.* (2022)	67 women with RPL	Endometrial biopsy	Midluteal phase	16S rRNA sequencing of V4 region	Not reported	Relative abundance Shannon index	*Ureaplasma relative abundance enriched in miscarriage*
Shu *et al.* (2022)	17 women with missed miscarriage 12 women requesting termination of normal pregnancy	Sheathed endometrial sampler	At the time of surgical procedure for miscarriage or termination	16S rRNA sequencing of V3–V4 region	Not reported	Shannon index Beta diversity of weighted and unweighted samples Relative abundance	*No difference in Shannon index but significant difference in beta diversity between groups.* *Proteobacteria and Firmicutes significantly elevated in missed miscarriage group*
Vomstein *et al.* (2022)	20 women with RPL 20 women with RIF 10 controls	Uterine flushing and suction retrieval of sample	Follicular phase, midcycle, luteal phase	16S rRNA sequencing of V3–V4 region	Negative controls used	Shannon index, Chao1 Relative abundance	*Longitudinal differences in diversity and composition across the menstrual cycle in RPL, RIF and controls.* *Reduced Lactobacillaceae in RPL group* *Diverse composition at family and genus level in RIF*
Wang *et al.* (2023)	38 women with missed miscarriage 18 women requesting termination of normal pregnancy	Sheathed endometrial sampler	At the time of surgical procedure for miscarriage or termination	16S rRNA sequencing of V4 region	Not reported	Chao1 Phylogenetic diversity Bray–Curtis Unweighted UniFrac index	*No difference in diversity between groups.* *Lactobacillus jensenii significantly different between groups but direction not specified.*

ITS-1, internal transcribed spacer 1; PERMANOVA, permutational multivariate analysis of variance; RIF, recurrent implantation failure; RPL, recurrent pregnancy loss; Vx, variable region.

An example of this literature is a recent study by Wang *et al.* (2023), which assessed the endometrial microbiota in patients with a missed miscarriage and reported no significant difference in either Chao1 alpha or Bray–Curtis beta diversity in comparison to those with a normal pregnancy. However, a significant difference in the abundance of *L. iners* (*P* < 0.05) was demonstrated between the two groups. Unfortunately, appropriate negative controls (e.g. kit reagent and environmental controls) were not included in this study and several of the species reported in the endometrium are common reagent contaminants (e.g. *Ralstonia* (Salter *et al.*, 2014)) or plant-associated bacteria (e.g. *Ensifer* and *Pseudarthrobacter*) that have rarely been genuinely identified in human samples.

Negative reagent controls were used by Peuranpää *et al.* (2022) who conducted a nested case-controlled study of the vaginal and endometrial microbiota of 47 women with a history of two or more early pregnancy losses and 39 healthy controls. Overall, strong inter-individual correlation between the vaginal and endometrial microbiota compositions was observed (*R* = 0.85, *P* < 0.001). Compared to healthy controls, women with RM had a significantly reduced relative mean abundance of *L. crispatus* (17.2% versus 45.6%, respectively, false discovery rate corrected *P* = 0.04) and increased levels of *Gardnerella vaginalis* (12.4% versus 5.8%, respectively, false discovery rate corrected *P* < 0.001). However, these findings were not replicated in a study by Vomstein *et al.* (2022) who assessed uterine aspirates from 20 women with a history of RM compared to 10 healthy controls and 20 women with recurrent implantation failure at IVF. No differences in Firmicutes, the phylum of which Lactobacilli are a member, or other measures of bacterial composition were seen in RM compared to controls, however, an increase in Proteobacteria in the RM and recurrent implantation failure group towards the end of the menstrual cycle was observed indicating the potential importance of temporal assessment of the endometrial microbiota.

Women with coeliac disease are enriched for the HLA-DQ2/DQ8 haplotype, which is also more prevalent in the RM populations (D'Ippolito *et al.*, 2016). Coeliac disease has been associated with alterations in the intestinal microbiota, thought in part to be mediated by HLA genes (Akobeng *et al.*, 2020). This led to the hypothesis that modulation of microbiota-host interactions mediated by HLA type may be driving RM. A recent study assessing the endometrial microbiota in women with a history of coeliac disease and RM found that women with a history of RM have reduced levels of vaginal and endometrial *Lactobacillus acidophilus* and increased levels of *L. iners* compared to controls, regardless of HLA haplotype (Masucci *et al.*, 2023). Although no microbial differences associated with HLA-DQ2/DQ8 haplotype were identified in sub-analyses of women with RM, these results indicate a potential interaction between host genetics and microbiota composition that may predispose women to an unfavourable endometrial microenvironment.

While studies have identified differences in the constitution of the endometrial microbiota in relation to miscarriage, it is conceivable that changes to microbiota composition across the cycle may precipitate miscarriage. Further work is required to assess the importance of temporal dynamics of the endometrial microbiota.

## Establishing causality between the endometrial microbiome and RM

To date, most evidence linking the endometrial microbiota with early pregnancy loss is associative. Whether reported relationships are causal or a consequence of physiological changes leading to miscarriage remains to be elucidated. However, emerging data from studies assessing host–microbial interactions in the context of early pregnancy are largely consistent with emergent theories on the mechanisms underpinning miscarriage.

The commensal endometrial microbiome may have a symbiotic relationship with the endometrium and its local immune mediators, facilitating implantation and modulating immune tolerance. The maternal immune system is imperative to early pregnancy success. Immune-mediated trophoblast invasion and spiral artery remodelling are key to achieve successful implantation (Brosens *et al.*, 2022). In addition, immunomodulation of endometrial senescence may further result in changes in the microenvironment predisposing towards pregnancy loss (Brighton *et al.*, 2017; Lucas *et al.*, 2020). It is plausible that microbial interaction with these key inflammatory pathways might impact endometrial homeostasis precipitating pregnancy loss.

**Figure 2.**
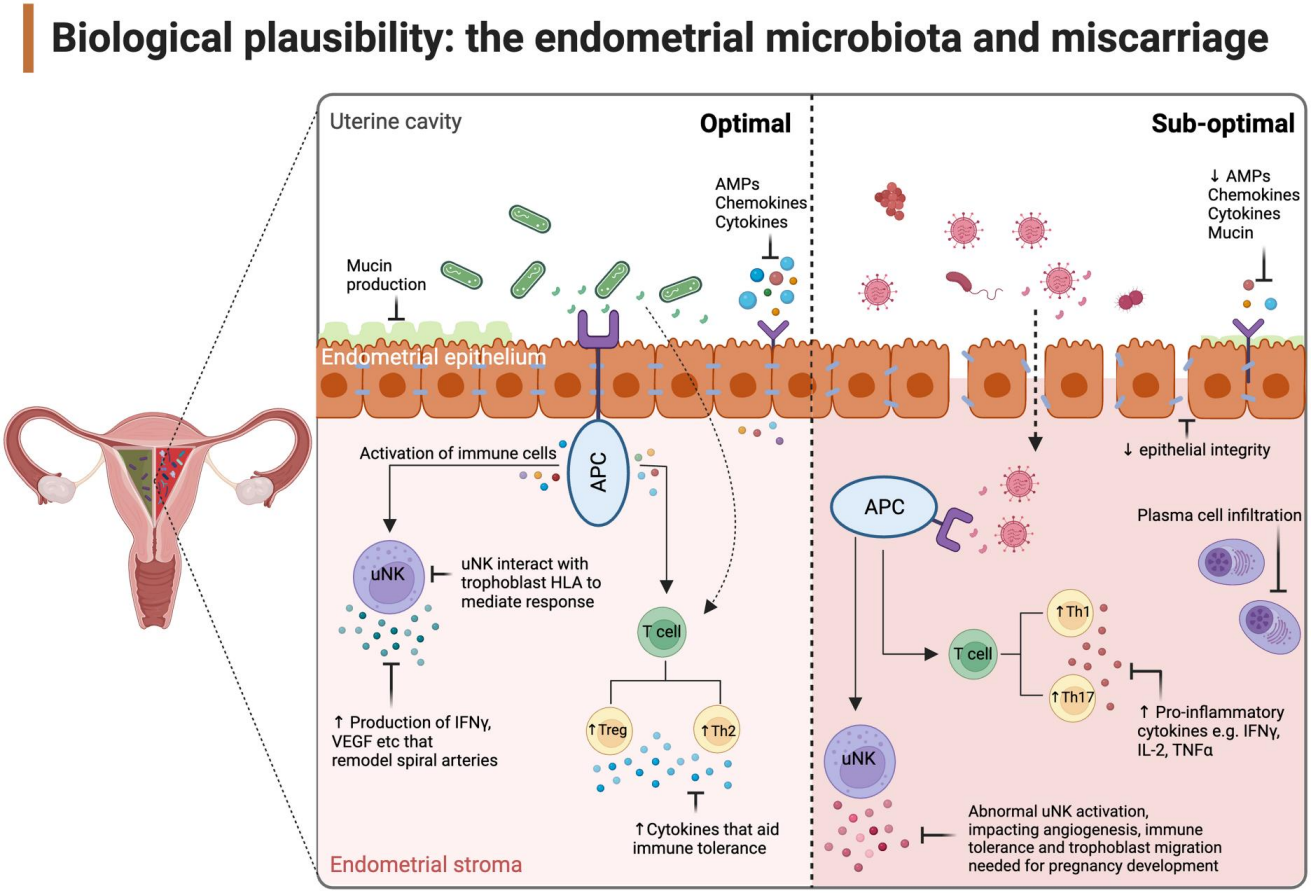
**Overview of potential mechanisms linking endometrial microbiota to early pregnancy loss.** In an *optimal* state, low levels of commensal bacteria promote host immune and inflammatory response supportive of endometrial function. Epithelial cells produce anti-microbial peptides (AMPs), chemokines and cytokines, helping to maintain epithelial integrity and repel potential pathogenic bacteria (Yarbrough *et al.*, 2015). Commensal bacteria and their metabolites interact with antigen-presenting cells (APCs) to modulate immune tolerance including the formation of specific T cells, such as regulatory T cells (Treg), and the transformation of Th1 to Th2 cells (Tsuda *et al.*, 2019; Zhu *et al.*, 2022). Th2 cells secrete IL-4, IL-6, IL-10, and IL-13, which are important for cytokine haemostasis and pathogen resistance (D'Ippolito *et al.*, 2018; Tsuda *et al.*, 2019) whereas uterine natural killer cells (uNK) interact with trophoblast HLA to modulate immune tolerance and secrete interferon (IFN)-γ and vascular endothelial growth factor (VEGF), which are involved in spiral artery remodelling for normal placentation(Brosens *et al.*, 2022; Faas and de Vos, 2017; Papúchová *et al.*, 2019). A shift towards a dysbiotic endometrial microbiota and increased pathogen colonisation could reduce AMP, chemokine, and cytokine production resulting in loss of epithelial integrity, increased permeability, and bacterial translocation (Benner *et al.*, 2018). Abnormally activated APCs drive aberrant immune pathway activation, including changes to T cell production leading to an increased ratio of Th17 and the transformation of Th2 to Th1 cells, leading to increased tumour necrosis factor (TNF)-α production, which has been implicated in abnormal pregnancy development (D'Ippolito *et al.*, 2018). Altered stimulation of uNK may affect angiogenesis and foetal–maternal immune tolerance (Brosens *et al.*, 2022) and plasma cells in the endometrial stroma may reflect an immune response to pathogens and impair endometrial receptivity (Buzzaccarini *et al.*, 2020; Teng *et al.*, 2012). Created with BioRender.com.

Evidence supporting microbiota-mediated dysregulation of inflammatory pathways in the endometrium in RM comes from a recent study comparing endometrial biopsy and endometrial lavage microbiota profiles in women with a history of RM (n = 25) and healthy controls (n = 25). Beta-diversity was found to be significantly higher in endometrial lavage fluid samples but not tissue biopsies of patients experiencing RM (Liu *et al.*, 2022). Concomitant changes in the inflammatory profile of these patients were seen, with significantly lower relative expression of interferon (IFN)-γ and IL-6 compared to controls (*P* = 0.013 and *P* = 0.038, respectively). The authors hypothesized that this may reflect RM-associated changes in the mucosal surface microbiota but not within tissue (Liu *et al.*, 2022). Changes in tissue inflammatory mediators, including increased levels of IL-6, IL-1β, heat-inducible factor-1α, and cyclo-oxygenase-2, have also been associated with endometrial dysbiosis in women with repeated implantation failure (Cela *et al.*, 2022). Although implantation is an inflammatory event, excessive inflammation outside of the implantation window has been associated with reproductive failure, including RM (Christiansen *et al.*, 2006). Altered endometrial inflammation is also a feature of chronic endometritis (CE), a possible cause of RM (Cicinelli *et al.*, 2014). In a study of the endometrial microbiota in infertile women with and without a history of CE a significant reduction in Lactobacilli relative abundance in women with CE was demonstrated (1.89% versus 80.7%, respectively; *P* = 0.034) (Liu *et al.*, 2019). Separate studies have also demonstrated CE to be associated with a significant decrease in alpha-diversity within the endometrial microbiota (Chen *et al.*, 2021) and increased expression of pro-inflammatory genes associated with apoptotic pathways, including IFN-γ and tumour necrosis factor-α (Cicinelli *et al.*, 2021). Interestingly, the relationship between endometrial microbial dysbiosis and increases in local inflammatory cytokines was not observed in women undergoing hysterectomy for benign gynaecological conditions (Mitchell *et al.*, 2015). These findings collectively infer that individual-level host response to alterations in the endometrial microbiota may be key determinants of specific microbiota-associated adverse outcomes in early pregnancy. A proposed mechanism by which the endometrial microbiota could influence key stages of early development is presented in Fig. 2. The peri-implantation endometrium has robust antimicrobial defences to protect the emergent conceptus. This includes an abundance of immune cells and expression of anti-microbial factors such as chemokine ligand 14 (Lea and Sandra, 2007). These factors may be suppressed in an aberrant endometrial environment potentially predisposing to the abnormal microbiota rather than being driven by it. Future work needs to further evaluate directionality of causation.

Despite uncertainty in the causal relationship between the endometrial microbiota and early pregnancy loss, there is some evidence that the endometrial microbiota may be predictive of RM. In a recent cohort study, 67 women with two or more previous miscarriages had endometrial biopsies taken outside of pregnancy (Shi *et al.*, 2022). Of these, 44 women subsequently became pregnant with 30 live births reported. Multivariate regression analysis of microbiota profiles showed that subsequent miscarriage was associated with increased relative dominance of *Ureaplasma* (odds ratio 24.2; 95% CI 1.55–377; *P* = 0.023). A similar predictive effect for the endometrial microbiota has been previously described in a study of 342 women with infertility in which a *Lactobacilli* enriched endometrial microbiota was found to be associated with increased probability of live birth (*Z*-score live birth: −0.12–1.51 versus clinical miscarriage: −2.81–0.55) (Moreno *et al.*, 2022). In this cohort, clinical miscarriage following IVF was associated with significantly higher relative abundance of *Haemophilus* and *Staphylococcus*. The study was, however, limited by a high sample failure rate further demonstrating the technical difficulties of low biomass microbial sampling. These studies tend to use a binary definition of RM, which may limit the conclusions drawn. Future work would benefit from an assessment of a gradation of risk, better mirroring the escalation in risk of miscarriage with increasing numbers of previous miscarriages seen clinically.

## Towards clinical translation

Current evidence linking the endometrial microbiota with RM and early pregnancy outcomes is predominantly associative. However, there is general agreement that displacement of low biomass *Lactobacillus* species from the endometrium by high diversity microbiota community compositions often enriched with pathogens is accompanied by an aberrant local immune response and inflammation. This could plausibly interfere with highly regulated physiological events critically involved in early development. Accordingly, strategies designed to treat sub-optimal endometrial microbial communities or prevent their formation could improve the endometrial microenvironment and reproductive outcomes. In support of this notion, it has recently been demonstrated *in vitro* that the probiotic *Lactobacillus* species, *Lactobacillus delbrueckii* and *Lactobacillus rhamnosus*, dampen lipopolysaccharide-induced expression of HLA-DR, CD86, CD80, CD83, and IL-12 from human dendritic cells (Esmaeili *et al.*, 2018), which may suggest a potential mechanism by which microbiota modulate immune-tolerance in early pregnancy (Inversetti *et al.*, 2023). Administration of a live vaginal biotherapeutic, LACTIN-V, containing *L. crispatus*, has also been shown to reduce the recurrence of bacterial vaginosis and urinary tract infections in non-pregnant women (Stapleton *et al.*, 2011; Cohen *et al.*, 2020) and recently was demonstrated to be safe and well tolerated in pregnant women (Bayar *et al.*, 2023). A large randomized controlled trial assessing the use of antibiotics in RM complicated by CE will shortly report on the effect of antibiotics on the endometrial microbiota (ISRCTN Registry, 2020). Such data will be informative in supporting or refuting causality between endometrial function and microbiota composition and guiding the potential treatment response of dysbiosis. To this extent, a recent proof of concept case-study reported that stable correction of vaginal dysbiosis using an antibiotic-free vaginal microbiota transplant in a patient with a history of RM was followed by a successful pregnancy and delivery (Wronding *et al.*, 2023). It remains, however, important to note that potentially confounding treatment with aspirin and low-molecular-weight heparin was also given within the successful pregnancy. Despite this, however, while larger studies are clearly required, this potentially offers an exemplar of how correction of the reproductive tract microbiota may eventually lead to improvement of reproductive health outcomes.

## Limitations of present literature

As highlighted in this review, a major limitation of the existing literature is its limitation to associative descriptions of the relation between miscarriage and the endometrial microbiota. It remains unclear as to whether changes in endometrial microbiota linked to miscarriage represent a cause or effect of the underlying endometrial changes pre-disposing to miscarriage. An improved understanding of this interplay is required to progress the field. The impact of contamination of the low-biomass endometrium at the time of sampling or during downstream analyses requires more consideration. Finally, high-quality randomized controlled trials are required to demonstrate the impact of microbiome-modulating interventions in the context of risk phenotypes associated with adverse early pregnancy outcomes.

## Conclusion

In conclusion, there is a growing body of evidence demonstrating an association between endometrial microbiota composition and early pregnancy loss, including RM. Causality, however, remains unproven. Further research is needed to determine factors that may predispose women to adverse microbiota–host responses and to enable identification and stratification of those who may benefit from interventions designed to modulate the reproductive tract microbiota.

## Data Availability

No new data were generated or analysed in support of this research.
